# Peculiarities of promiscuous l-threonine transaldolases for enantioselective synthesis of β-hydroxy-α-amino acids

**DOI:** 10.1007/s00253-021-11288-w

**Published:** 2021-04-26

**Authors:** Shan Wang, Hai Deng

**Affiliations:** Department of Chemistry, University of Aberdeen, Aberdeen, Scotland AB24 3UE UK

**Keywords:** l-threonine transaldolases (LTTAs), β-Hydroxy-α-amino acids (βHAAs), Stereoselective chemoenzymatic synthesis, Natural product biosynthesis

## Abstract

**Abstract:**

The introduction of β-hydroxy-α-amino acids (βHAAs) into organic molecules has received considerable attention as these molecules have often found widespread applications in bioorganic chemistry, medicinal chemistry and biomaterial science. Despite innovation of asymmetric synthesis of βHAAs, stereoselective synthesis to control the two chiral centres at C_α_ and C_β_ positions is still challenging, with poor atomic economy and multi protection and deprotection steps. These syntheses are often operated under harsh conditions. Therefore, a biotransformation approach using biocatalysts is needed to selectively introduce these two chiral centres into structurally diverse molecules. Yet, there are few ways that enable one-step synthesis of βHAAs. One is to extend the substrate scope of the existing enzyme inventory. Threonine aldolases have been explored to produce βHAAs. However, the enzymes have poor controlled installation at C_β_ position, often resulting in a mixture of diastereoisomers which are difficult to be separated. In this respect, l-threonine transaldolases (LTTAs) offer an excellent potential as the enzymes often provide controlled stereochemistry at C_α_ and C_β_ positions. Another is to mine LTTA homologues and engineer the enzymes using directed evolution with the aim of finding engineered biocatalysts to accept broad substrates with enhanced conversion and stereoselectivity. Here, we review the development of LTTAs that incorporate various aldehyde acceptors to generate structurally diverse βHAAs and highlight areas for future developments.

**Key points:**

• *The general mechanism of the transaldolation reaction catalysed by LTTAs*

• *Recent advances in LTTAs from different biosynthetic pathways*

• *Applications of LTTAs as biocatalysts for production of βHAAs*

## Introduction and scope of the review

**Fig. 1:**
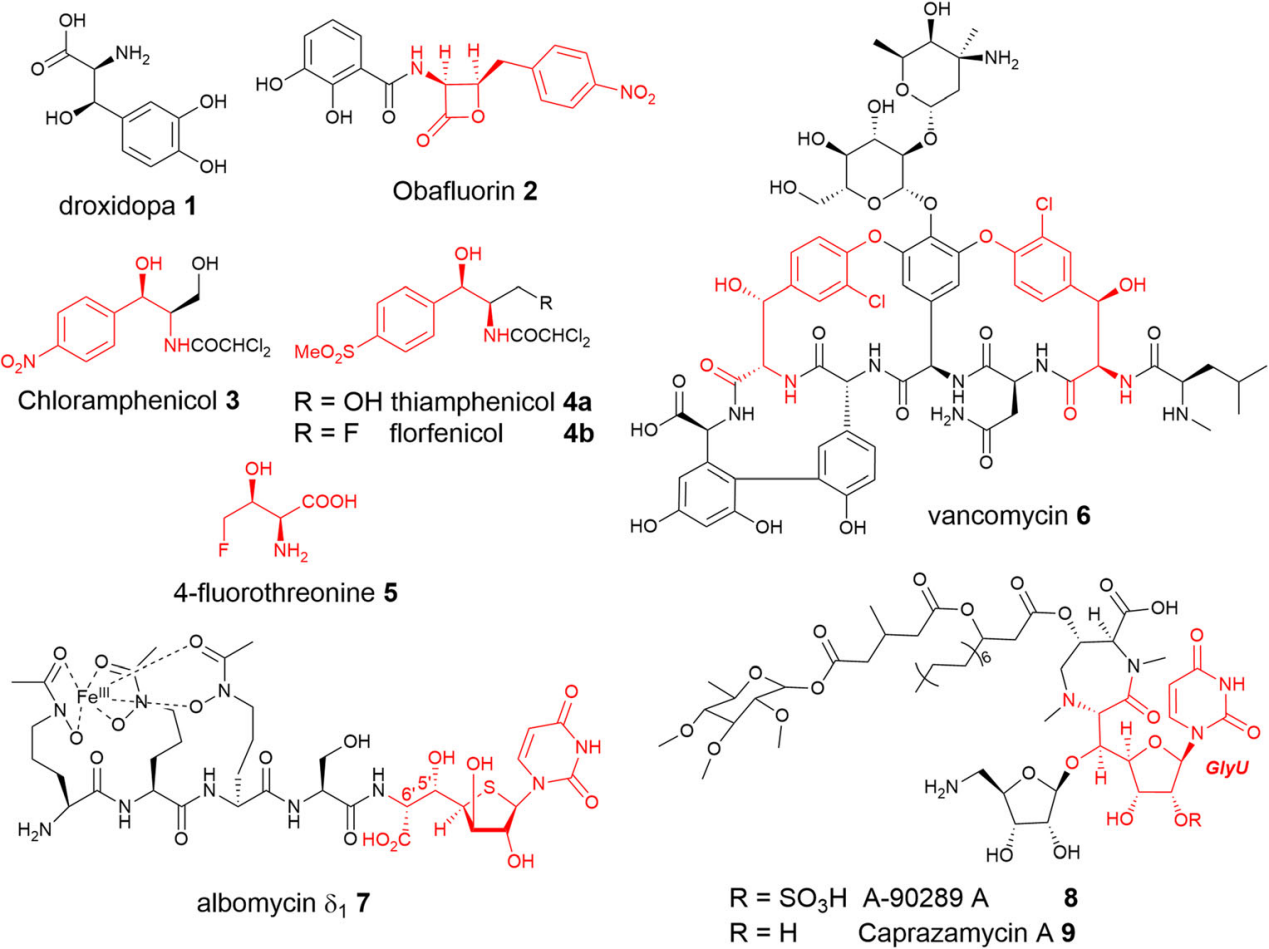
β-hydroxy-α-amino acids (βHAAs) serve as building blocks for many biological active chemicals and pharmaceutical drugs

Nature often employs non-canonical amino acids that bear new and different functional groups to tune the properties of bioactive small molecules (Hibi et al. 2015). There are dozens of known modifications to proteogenic amino acids, including a wide variety that bears a hydroxyl group at the β-carbon. β-Hydroxy-α-amino acids (βHAAs) are an important class of compounds, the majority of which contain two chiral centres at C_α_ and C_β_ position, resulting in four possible diastereoisomers (Ashford and Bew 2012). They are valuable constituents of medicinally important compounds and complex natural products (Hibi et al. 2015). For instance, l-*threo*-3,4-dihydroxyphenylserine (Droxidopa) **1** (Fig. 1) is a currently used therapeutic for the treatment of Parkinson disease (Maruyama et al. 1996; Hauser et al. 2016). βHAAs have also been used as intermediates in the biosynthesis of β-lactones such as obafluorin **2** (Fig. 1) (Schaffer et al. 2017; Scott et al. 2017). l-*threo*-*p*-nitrophenylserine and its derivative are the key components of amino alcohol antibiotics, chloramphenicol **3** (Seiple et al. 2014), its synthetic derivatives thiamphenicol **4a** and fluorfenicol **4b** and 4-fluorothreonine **5** (Fig. 1) (Lu et al. 2008); β-hydroxytyrosine and β-hydroxyphenylalanine residues are found in clinically active glycopeptide antibiotics, such as vancomycin **6** (Fig. 1) (Yim et al. 2014). Nucleotide-containing βHAA is the crucial motif of the nucleoside antibiotic natural products **7**–**9** (Fig. 1) (Barnard-Britson et al. 2012; Cai et al. 2015; Ushimaru and Liu 2019). βHAAs are also common synthetic precursors to β-lactams and aziridine carboxylic acid derivatives in synthetic chemistry and to chiral bisoxazoline ligands for metal catalysis (Masruri et al. 2012).

These diverse applications underlie a broad interest in understanding βHAA production and utilization in nature. Accordingly, several methods have been devised for the asymmetric syntheses of βHAAs including asymmetric aldol reaction (Sugiyama et al. 2002 and Singjunla et al. 2013), sharpless asymmetric dihydroxylations (Masruri et al. 2012), stereoselective electrosynthesis using *trans*-metal Schiff base complexes (Levitskiy et al. 2019) and numerous others. The main challenge is the control of the relative and absolute stereochemistry of the asymmetric carbons in the current trend of sustainable development.

Compared to non-enzymatic synthesis, an attractive alternative is enzymatic synthesis of βHAAs via a one-step process under mild conditions as a means of potentially more atom-economic and greener. There have been considerable attentions that are focused on those promising biotransformation strategies for asymmetric synthesis of βHAAs. Nature has evolved different enzymatic routes to produce these useful compounds. Free amino acids can undergo stereo-specific and -selective Cβ hydroxylation, as illustrated by the non-heme Fe-dependent hydroxylation of homotyrosine in the biosynthesis of echinocandins (Hüttel. 2021). Another common biosynthetic strategy is for an amino acid to be loaded onto a non-ribosomal peptide synthetase (NRPS), whereupon a P450 enzyme catalyses Cβ hydroxylation of an aminoacyl-*S*-enzyme intermediate en-route to a peptide natural product as observed in the biosynthesis of vancomycin **1** (Tailhades et al. 2020).

Threonine aldolases (TAs), which catalyse a reversible chemical reaction of cleavage of threonine into glycine and acetaldehyde, have been reported as such biocatalysts using various aldehydes and glycine as substrates to generate useful βHAAs (Fesko 2016). However, the poor enantioselectivity at C_β_ hampers their further application as side products are generated. In contrast, there is a remarkable set of pyridoxal-5′-phosphate (PLP)-dependent enzymes, l-threonine transaldolases (LTTAs), which catalyse retroaldol cleavage of l-threonine (Thr) and a subsequent aldol-like addition into an aldehyde to form a new side chain, setting the stereochemistry of the C_β_−OH group. It is worth noting that phylogenetic analysis suggested that LTTAs are evolutionary distinct from l-threonine aldolases (LTAs) and SHMTs (Scott et al. 2017; Wu et al. 2020).

This review surveys the recent advances of LTTAs discovered in the biosynthetic pathways as potential biocatalysts, highlighting the unique biochemical transformations and their substrate promiscuity. Particular emphasis is placed on exploring the existing LTTA inventory for the production of structurally diverse βHAAs. Furthermore, bioengineering of such enzymes will certainly advance our abilities to engineer a suitable biotechnologically viable catalyst for βHAA production. Other PLP-dependent enzymatic reactions discovered from natural product biosynthesis can be found in the recent review (Du and Ryan 2019). Another family of transaldolases from the pentose phosphate pathway, catalysing the reversible transfer of a dihydroxyacetone moiety between ketose donors and aldose acceptors (d-glyceraldehyde-3-phosphate, or d-erythrose-4-phosphate), was summarized in a review (Samland et al. 2011). Therefore, this group of transaldolases will be out of the scope in the current report.

Although the first LTTA, 4-fluorothreonine transaldolase (FTase), was identified in 2001 (Murphy et al. 2001), it was not until 11 years later in 2012 that a second LTTA was identified (Barnard-Britson et al. 2012). Since then, five new LTTA homologues have been discovered during the biosynthetic studies of various bioactive natural products, coincided with the advanced genome sequencing technology during the last decade. It is noteworthy that while FTases are didomain LTTAs, newly discovered LTTAs are single-domain enzymes. Automated annotation of the sequenced genomes has miscategorized many LTTA-like open reading frames (ORFs) in microbial genomes as serine hydroxymethyltransferase (SHMT)-like enzymes (Scott et al. 2017). However, these LTTA-like ORFs share low homologues (25–35% aa identity) to SHMTs. Therefore, new LTTA homologues with improved kinetics and broad substrate specificity could be identified and characterized through conserved genomic mining of biotechnological purpose for asymmetric synthesis of βHAAs. This review will serve as a compendium to communities of microbiologists, organic chemists, natural product chemists, biochemists, synthetic biologists, and many others interested in the subject.

## The general mechanism of LTTAs

**Fig. 2:**
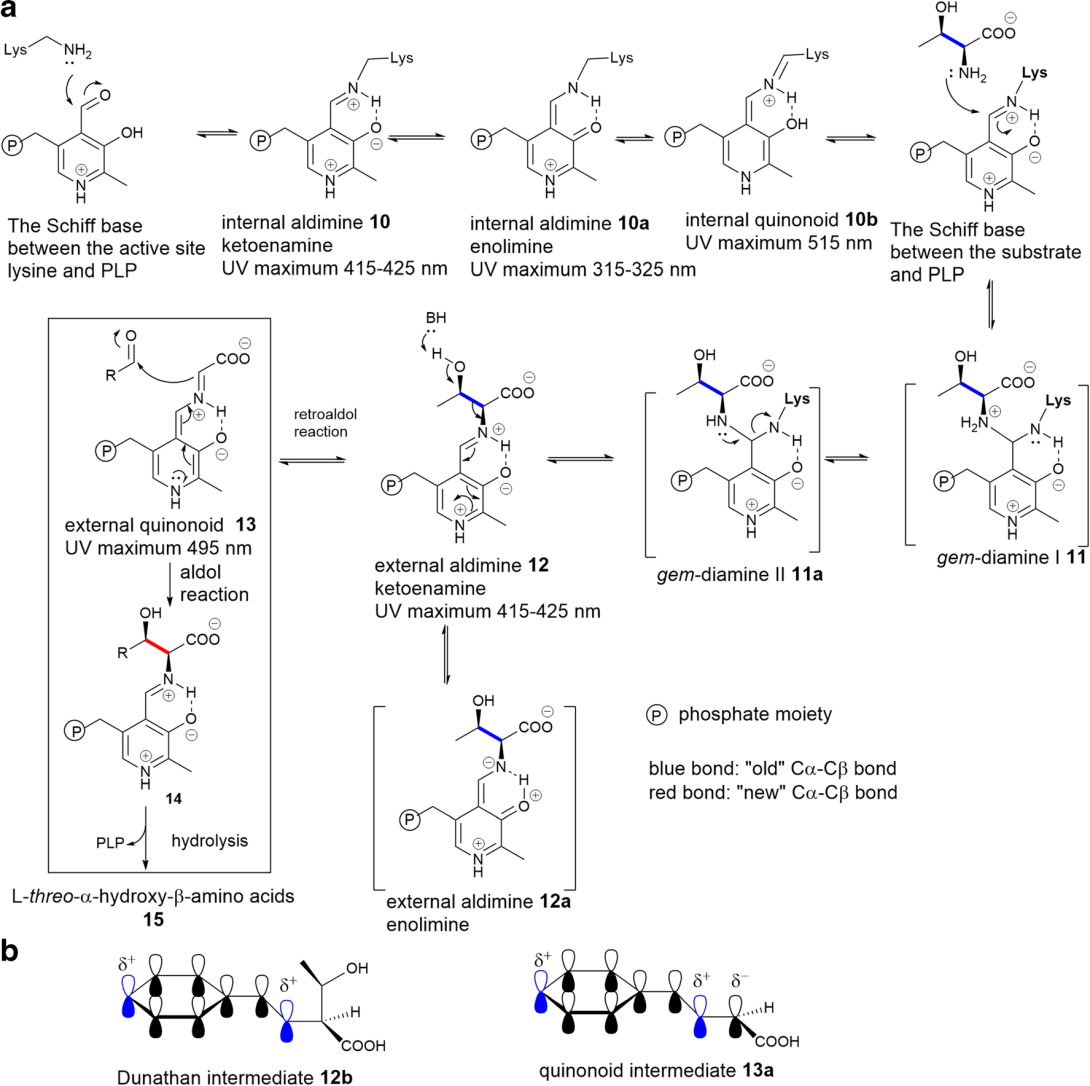
**a** A generic mechanism of the transaldolation reaction catalysed by LTTAs. **b** The schematic representation of Dunathan intermediate **12a** and quinonoid intermediate **13a**

All of the LTTA enzymes utilize PLP as the cofactor. The catalytic cycle starts and ends with the same PLP cofactor form (the internal aldimine **10**), which is derived from transamination and heterolytic cleavage to maintain the carbanionic intermediates (Richard et al., 2009). The internal aldimine structure consists of an imine linkage between the amino group of a lysine residue from the enzyme and the aldehyde carbon of free PLP. Then, within the interaction of l-Thr, a geminal diamine intermediate **11** is formed which finally leads to the PLP:external aldimine **12** allowing substrate binding and product release (Fig. 2). Additionally, the proton in the external aldimine can transfer (or is shared) between oxygen in the pyridine ring and imine nitrogen forming two tautomeric isoforms, enolimine **12** and ketoenamine **12a** separately. This transformation shows specific maximal absorbance values at 335 nm for enolimine **12a** and 425 nm for ketoenamine **12** when analysed by ultraviolet-visible (UV-Vis) spectroscopy.

The PLP:external aldimine **12** is the crucial intermediate for all LTTAs. Meanwhile, the efficiency of the PLP enzymatic reaction is predominately dependent on the heteroaromatic pyridine ring. The electrophilicity of the C4 of the external aldimine is enhanced because of the protonated pyridinium nitrogen, the resonance and hydrogen bonding of the hydroxyl group, leading to an ‘electron sink’. Therefore, the protonated imine nitrogen can withdraw electrons from the substrate. The π-conjugation of the pyridine ring delocalizes and stabilizes the net-negative charge of external aldimine. Due to the electronic delocalization energy, the stereochemical rearrangement of σ bonds is activated by a π system, forming the Dunathan intermediate **12b** (Fig. 2b). The deprotonation of hydroxyl group at Cβ of l-Thr residue results in a retroaldol cleavage between the C_α_-C_β_ bond, promoting the bond-breaking process of the perpendicular bond of the pyridine ring **13a** (Fig. 2b). As a consequence, the heterolytic cleavage happens, and the C_α_ hybridization changes generate the resonance-stabilized ubiquitous quinonoid intermediate **13** which displays specific maximal absorbance values at 495 nm. However, the quinonoid intermediate has not always been observed by UV-Vis spectroscopy in many the PLP-dependent reactions, suggesting that they may be extremely short lived or transient. A subsequent aldol-like addition into an aldehyde forms a new side chain **14**, setting the stereochemistry of the Cβ-OH group to yield new l-threo-α-hydroxy-β-amino acids **15** after hydrolysis (Fig. 2a). It is worth noting that LTTAs are evolutionarily divergent from TAs as such the first half of the biochemical pathway of LTTAs processes a retroaldol reaction, significantly different from TAs.

## Discovery of l-Thr transaldolases from the biosynthetic pathways of natural products

### The discovery of the didomain 4-fluorothreonine transaldolases (FTases)

**Fig. 3:**
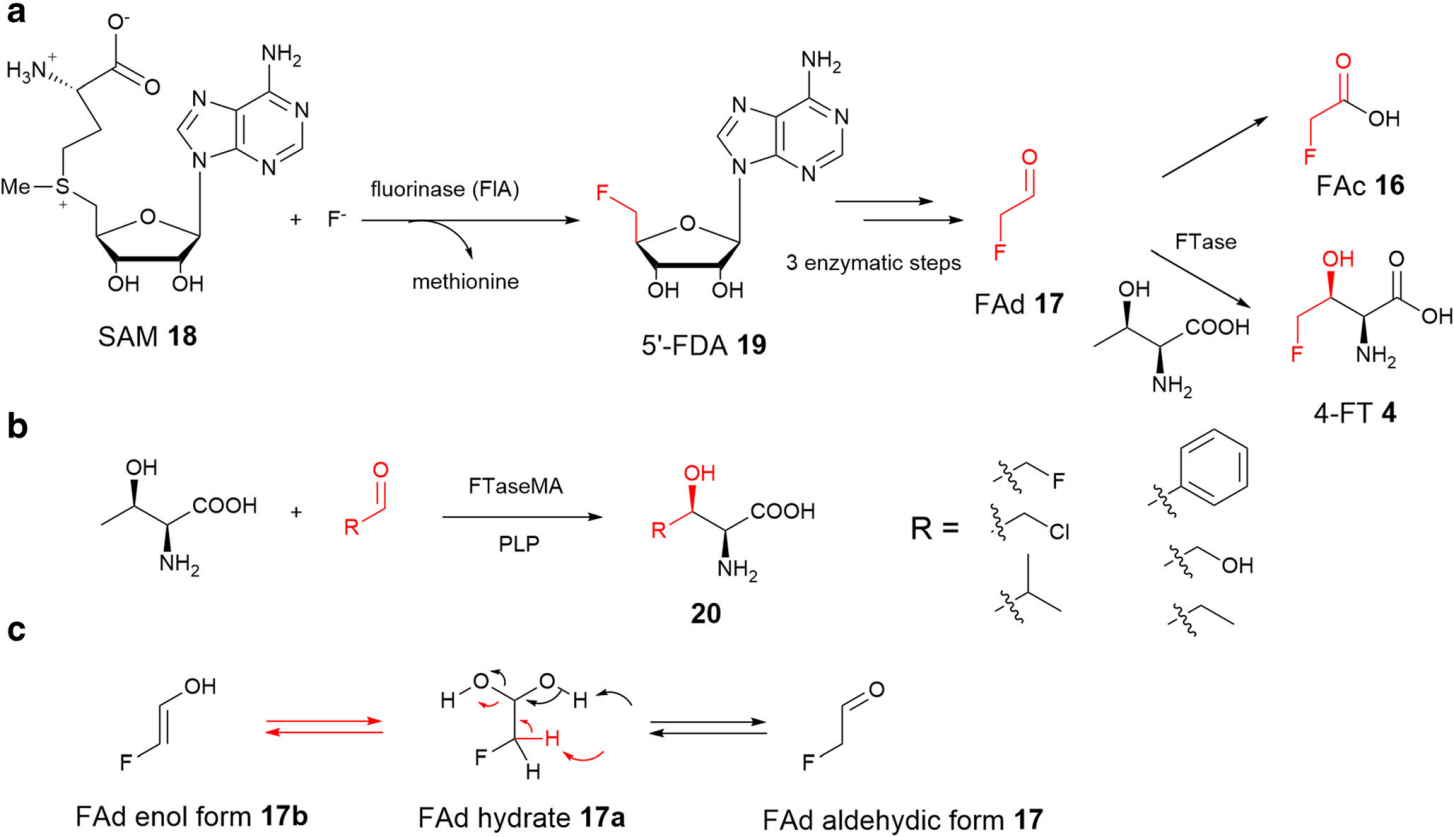
**a** The biosynthetic pathways of 4-fluorothreonine and fluoroacetate, highlighted with the first committed enzymatic step in the pathway of the fluorination enzyme. The fluorinase (FlA) in *Streptomyces cattleya* and *Streptomyces* sp. MA37 (MA37). **b** FTaseMA from MA37, the close homologue of the original FTase identified in *S. cattleya*, displays broad substrate specificity toward various aldehyde acceptors. **c** The proposed mechanism of the dehydration of FAd hydrate **17a** to FAd aldehydic form **17** catalysed by the second small domain of FTaseMA for the transaldolation reaction

Fluorine has emerged as a privileged element in medicinal chemistry as well as in agrochemistry and materials science (O’Hagan and Deng 2015). Fluorine substitutions are now considered a standard strategy for modulating the properties of chemical leads, despite that environmental contamination by organofluorines is an issue of major concern due to their persistence to biodegradation (Moreira et al. 2018). While many chemical methods of selective synthesis of organofluorines have been developed, it is attractive to consider biotransformation approaches to selectively access novel organofluorine chemicals, rather than using challenging chemical methods (Wu et al. 2020). Fluorinated NPs are rare, mainly due to the highest heat hydration of fluoride in water which makes fluoride a poor nucleophile. As a result, there are only a handful of fluorinated natural products discovered by organic chemists in natural product inventory. 4-Fluorothreonine (4-FT) **5** is the only fluorinated amino acid natural product discovered thus far. It was found to be a co-occurrent metabolite with another fluorinated toxic, fluoroacetate (FAc) **16**, in the culture broth of the soil bacterium *Streptomyces cattleya* in 1986 (Fig. 3a) (Sanada et al. 1986). O’Hagan and co-workers have conducted the seminal work on elucidation of the fluorometabolism in *S. cattleya* (Deng et al. 2004). They demonstrated that fluoroacetaldehyde (FAd) **17** is the precursor of 4-FT (Hamilton et al. 1997; Nieschalk et al. 1997; Moss et al. 2000), but glycine is not directly contributed to the 4-FT biosynthesis (Hamilton et al. 1998) in their early isotopic labelling studies, suggesting that the biotransformation of FAd **17** to 4-FT **5** is not descended from a classical aldolase reaction. In 2001, the enzyme, 4-fluorothreonine transaldolase (4-FTase), responsible for the production of 4-FT, was purified from *S. cattleya* (Murphy et al. 2001). Biochemical analysis using a combination of isotopic labelling experiments and GC-MS analysis demonstrated that this wild-type enzyme actually catalyses a PLP-dependent cross-over transaldolation reaction between FAd and l-Thr to give 4-FT and acetaldehyde as shown in Fig. 3a. The hallmark of the fluorometabolism studies is the discovery of the first native fluorination enzyme, fluorinase (FlA), which catalyses C-F bond formation between fluoride ion and *S*-adenosyl-l-methionine (SAM) **18** to generate 5′-fluoro-5′-deoxyadenosine **19** (O’Hagan et al. 2002; Dong et al. 2004). **19** is the first committed fluorinated intermediate in the 4-FT and FAc biosynthetic pathway. Sequence analysis of the cosmid DNA containing the *fl*A gene indicated that the gene-encoded FTase is not in the close proximity of *fl*A (Huang et al. 2006). To identify and clone the gene encoding FTase, degenerate PCR primers were designed based on MS-MS amino acid sequencing of FTase purified from *S. cattleya*. Subsequent chromosomal walking strategy allowed identification of a 1905 bp open-reading frame (*orf*). The encoded gene product contains 634 amino acids (aa), composed of two domains. The larger domain (S-domain) in the *N*-terminal shares ~44% sequence identity with SHMTs from archaea and thermophilic bacteria, while the smaller one (A-domain) in the C-terminal displays homology (~28% aa identity) with bacterial ribulose-1-phosphate-4-epimerase (Deng et al. 2008). It appears that FTase has a hybrid construction with key binding motifs from these distinct enzymes. The *flFT* gene was introduced into the model strain, *S. lividans* TK24, for overexpression. Although the enzyme expression level was low, incubation of recombinant enzyme with l-Thr and FAd **17** generated 4-FT **5** as monitored in ^19^F-NMR. *In vitro* pathway reconstitution indicated that fluoride ion can be converted to 4-FT involving five overexpressed enzymes, including recombinant fluorinase and FTase (Deng et al. 2008). Inactivation of *flFT* in *S. cattleya* resulted in the sole production of fluoroacetate (Zhao et al. 2012), further confirming the role of FTase in 4-FT biosynthesis.

The genome sequencing efforts of the second decade of the twenty-first century have revealed that another four actinomycete strains encode the *flA* genes, suggesting that these four new strains may have capacity to produce fluorometabolites (Deng et al. 2014; Huang et al. 2014; Wang et al. 2014). Of particular relevance here is the strain isolated from a Ghanaian soil sample, *Streptomyces* sp. MA37. In culture, the organism had the capacity to biosynthesise 4-FT **5** and FAc **16**, as well as a range of other minor fluorometabolites (Ma et al. 2015; Wu et al. 2020). The *flFT* homologues encoding the last step of 4-FT biosynthesis are in close proximity to the corresponding *flA* homologue, in line with expectation. This is not the case for *S. cattleya*, where *flFT* is located on the megaplasmid pSCATT (1.8 Mbp in length) and the *flA* gene is on the chromosome of *S. cattleya* (Zhao et al. 2012).

More recently, the overexpression of *flFT* homologous gene (*flFTMA*) from *Streptomyces sp.* MA37 in different hosts was carried out for detailed biochemical analysis (Wu et al. 2020). Among three *Streptomyces* hosts (*S. lividans* TK24, *S. lividans* 66 and *S. albus* J-1074), the *S. lividans* 66 recombinant variant gave the highest production of 4-FT while the least activity was found in *S. lividans* TK24 recombinant strain. The recombinant protein (FTaseMA) was purified to near homogeneity as a bright yellow protein with UV absorbance maxima at 335 and 425 nm, in which the enolimine peak at 325 nm is dominant. The stoichiometry between bound PLP and the protein was estimated to be 1:1. Incubation of the purified recombinant enzyme with l-Thr, FAd and PLP resulted in the production of 4-FT **5**, demonstrating that the recombinant protein is functional (Wu et al. 2020).

FTases are unusual enzymes in that they use FAd **17** as a substrate, which is heavily hydrated in water as FAd hydrate **17a** (Fig. 3c) (Cobb et al. 2004). As a result, the enzymes appear to evolve specific ability of accelerating the transformation of FAd hydrate **17a** to the aldehyde form **17** or FAd enol form **17b** for the next transaldolation reaction (Fig. 3c). This is in line with the hybrid construction of functional FTases that have a small domain (A domain) homologous to aldolase/epimerase possibly involving the dehydrate reaction. Structural prediction of this A domain suggested good structural similarity with the metal-dependent class II aldolases (Wu et al. 2020). The active sites of these aldolases normally contain a catalytic tetrad of Glu-His-His-His, the latter three His residues of which coordinate a divalent metal acting as a Lewis acid type catalyst. The general mechanism of these aldolases involves the first Glu residue directing proton transfer of the aldol to generate an enediol intermediate, coordinated by the bound zinc. Examination of the predicted structure of the aldolase/epimerase domain of FTaseMA suggested a catalytic tetrad, Glu573-His551-His553-His598, with the essential residue of Asn484 that may position Glu573 residue towards the active site through hydrogen bonds. Indeed, elemental analysis of FTaseMA demonstrated that the stoichiometry of the bound zinc and the enzyme was estimated to be 1:1 (Wu et al. 2020). Alanine-scanning mutagenesis of the three His and Asn residues significantly reduced the production of 4-FT **5**, suggesting that these four residues are important (Wu et al. 2020). The mutated FTaseMA variant, in which Glu573 was changed to Ala, however, caused the instability of the purified protein for further biological evaluation.

FTaseMA displays considerable substrate plasticity. Although FTaseMA cannot accept d-*allo*-threonine, d-threonine or glycine or alanine or leucine as substrates, consistent with the previous studies of FTase from *S. cattleya* (Murphy et al. 2001), it can use L-Ser as substrate, albeit with low conversion of 4-FT as monitored in ^19^F-NMR. Most of the characterized LTTAs were found to accept L-Thr as the only retroaldol donor (Wu et al. 2020). Strikingly, the enzyme can also utilize L-*allo*-Thr as substrate with nine-fold less efficiency than the natural substrate l-Thr, a clear difference compared to other LTTAs. FTaseMA also accepts a range of electrophilic aldehyde acceptors with different functionalities, such as chloroacetaldehyde, glycolacetaldehyde, propanal, isobutyraldehyde, phenylacetaldehyde, when l-Thr was used, to generate different βHAAs, suggesting that this enzyme could be used as a potential versatile biocatalyst to produce various βHAAs (Wu et al. 2020). Of particular interests here is glycolacetaldehyde which is heavily hydrated in water. Seventy per cent of glycolacetaldehyde in the aqueous solutions exists as a diol form while only 4% is the aldehyde form (Collins and George 1971), further demonstrating the unusual ability of FTaseMA to dehydrate the diol of hydrated aldehyde species.

### LTTAs from the biosynthetic pathways of nucleoside antibiotic natural products

**Fig. 4:**
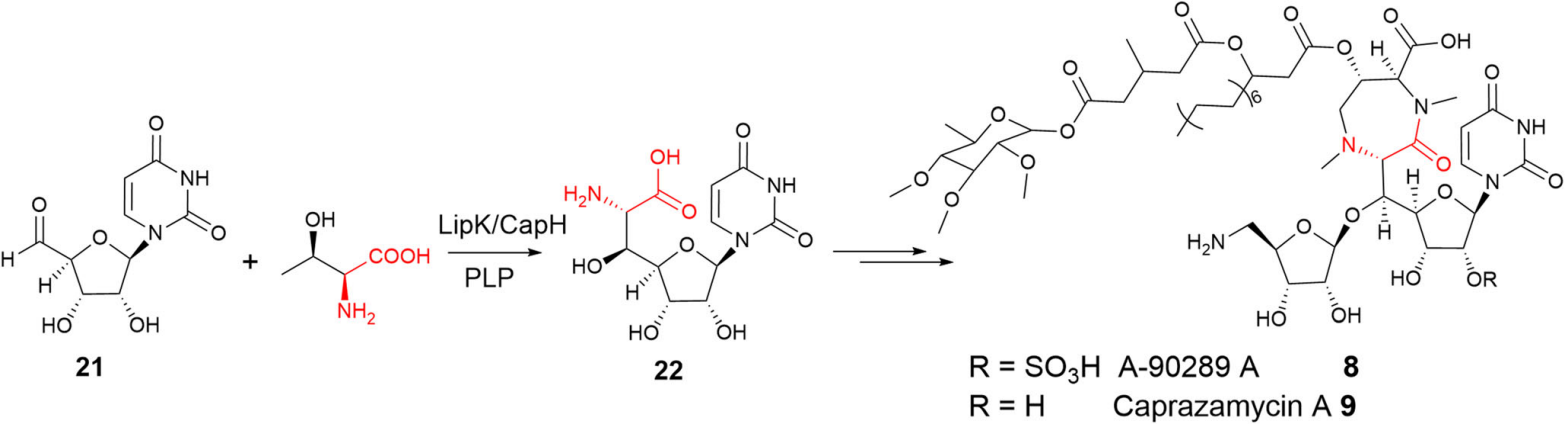
LipK and CapH are two LTTAs catalysing the transaldolation between l-Thr and uracil 5′-aldehyde **21** in the biosynthetic pathways of lipopeptidyl nucleoside antibiotic natural products

Several lipopeptidyl nucleoside antibiotics, such as A-90289s **8** from *Streptomyces* sp. SANK 60405 (Fujita et al. 2011), and caprazamycins **9** from *Streptomyces sp*. MK730-62F (Igarashi et al. 2005), contain highly modified furanose motifs (Figs. 1 and 4). Previous isotopic labelling studies suggested that a C–C bond forming event, possibly via an aldol mechanism, occurs extending the carbon chain at C-5′ of the canonical nucleoside to C6 or longer chain length of a so-called high-carbon nucleoside scaffold (Winn et al. 2010).

With the advance of genome sequencing technology in the last decade, the biosynthetic gene clusters (BGCs) of these nucleoside antibiotics have been identified for characterization (Barnard-Britson et al. 2012). Bioinformatics analysis of these clusters revealed that they shared an *orf*, putatively annotated as SHMT-like enzyme. This analysis was in line with the prediction of an aldol reaction that the putative SHMT ORFs may catalyse a biotransformation of Gly and nucleoside 5′-aldehyde **21** to yield the corresponding high-carbon nucleoside intermediates during the biosynthesis of these antibiotics.

Heterologous expression of *lip*K, encoding a putative SHMT in the BGC of A-90289s in *S. lividan* TK24, resulted in a soluble recombinant protein which contains a characteristic UV absorption maximum of 415 nm, corresponding to a typical *holo* PLP-dependent enzyme (Fig. 2a). This UV absorption was abolished in a LipK variant where Lys235 was changed to Ala, demonstrating that Lys235 is the key aa residue for the catalytic cycle (Barnard-Britson et al. 2012).

Tests on the activity of LipK with **21** and Gly or other l-amino acids revealed that only incubation of l-Thr resulted in the appearance of a new compound as observed in HPLC analysis. The identity of the new compound was confirmed to be the βHAA containing glycine uracil **22** as evidenced by high-resolution MS and comprehensive 1D and 2D NMR analysis, in comparison to synthetic materials. The absolute stereochemistry of **22** was determined to be (5′*S*, 6′*S*) through the diagnostic *J* coupling value (*J*_H6′-H5′_ = 5.0 Hz) of the derivatised synthetic and enzymatic **22**. The enzyme displays restricted substrate specificity and only accepts l-Thr as the substrate. Unlike FTases, LipK is a single-domain LTTA. Further conserved genomic analysis indicated the occurrence of LipK homologues, suggesting that l-Thr transaldolation reaction may be more common than previously anticipated. LipK represents the second confirmed LTTA and the first subgroup of l-Thr:uridine-5′-aldehyde (UA) transaldolase to catalyse the biotransformation of βHAA-containing nucleosides, providing a new biotechnological platform to prepare βHAA-containing sugar previously difficult to be synthesized.

The second LipK-like LTTA, CapH, was identified in the BGC of capuramycin-type antibiotics A-500359s (Cai et al. 2015). Characterization of recombinant CapH revealed that CapH displays a similar biochemical property compared to LipK with restricted substrate specificity towards l-Thr. It has remained to be determined whether LipK or CapH utilises other nucleotide aldehydes except UA.

### LTTA from the pathway of β-lactone antibiotic

The antibiotic natural product obafluorin **2** (Fig. 1) belongs to the chemical class of β-lactones, consisting of strained four-membered rings, closely related to β-lactam family of antibiotics, such as penicillin. Obafluorin **2** contains an interesting βHAA motif, (*R*)-β-hydroxy-*p*-nitro-l-homophenylalanine (β-OH-*p*-NO_2_-homoPhe) **23**. In 2017, the corresponding BGC (*oba*/*obi*) of obafluorine **2** was identified in the producer, *Pseudomonas fluorescens*, by two research groups simultaneously (Schaffer et al. 2017; Scott et al. 2017). Bioinformatics analysis suggested that one of the *orf*s in the BGC encodes a putative bacterial SHMT albeit low aa identity (25%). Overexpression of *obi*H (*oba*G) in *E. coli* resulted in the purification of the recombinant protein with unusual pink colour (Scott et al. 2017; Kumar et al. 2021). Biochemical assays coupled with isotopic labelling experiments demonstrated that ObiH (ObaG) is a new LTTA that catalyses the transaldolation between l-Thr and *p*-nitro-phenylacetaldehyde **24** to generate a single stereoisomer of β-OH-*p*-NO_2_-homoPhe with a yield of 55–59% (Scott et al. 2017).

ObiH (ObaG) displays a UV/Vis spectrum characteristic of PLP-dependent proteins, comprising absorption maxima at 340 nm and 390 nm that correspond to the internal ketoenamine **10** and enolimine **10a** (Fig. 2a), respectively (Scott et al. 2017). The pink colour of ObiH attributing the UV absorption at 512 nm is correlated to the unusual internal quinonoid **10b** (Fig. 2a), which is inactive state of the enzyme (Kumar et al. 2021). Exposing ObiH in the UV led to the rapid photoablation of the 515-nm peak and a temporary increase at 340 nm, followed by decrease of 340 nm band and increase of 415 nm band, indicative of an isomerization between the enolimine **10a** and ketoenamine **10** of the cofactor, respectively. Addition of l-Thr led to the formation of external glycyl quinonoid intermediate **13** that is remarkably persistent with a half-life of ~3 h, suggesting that ObiH catalyses the retro-aldol cleavage of l-Thr in the reaction cycle. The long lifetime of this glycyl quinonoid intermediate **13** would enable the enzyme reactivity with non-native aldehyde substrates for potential biocatalyst development.

**Fig. 5:**
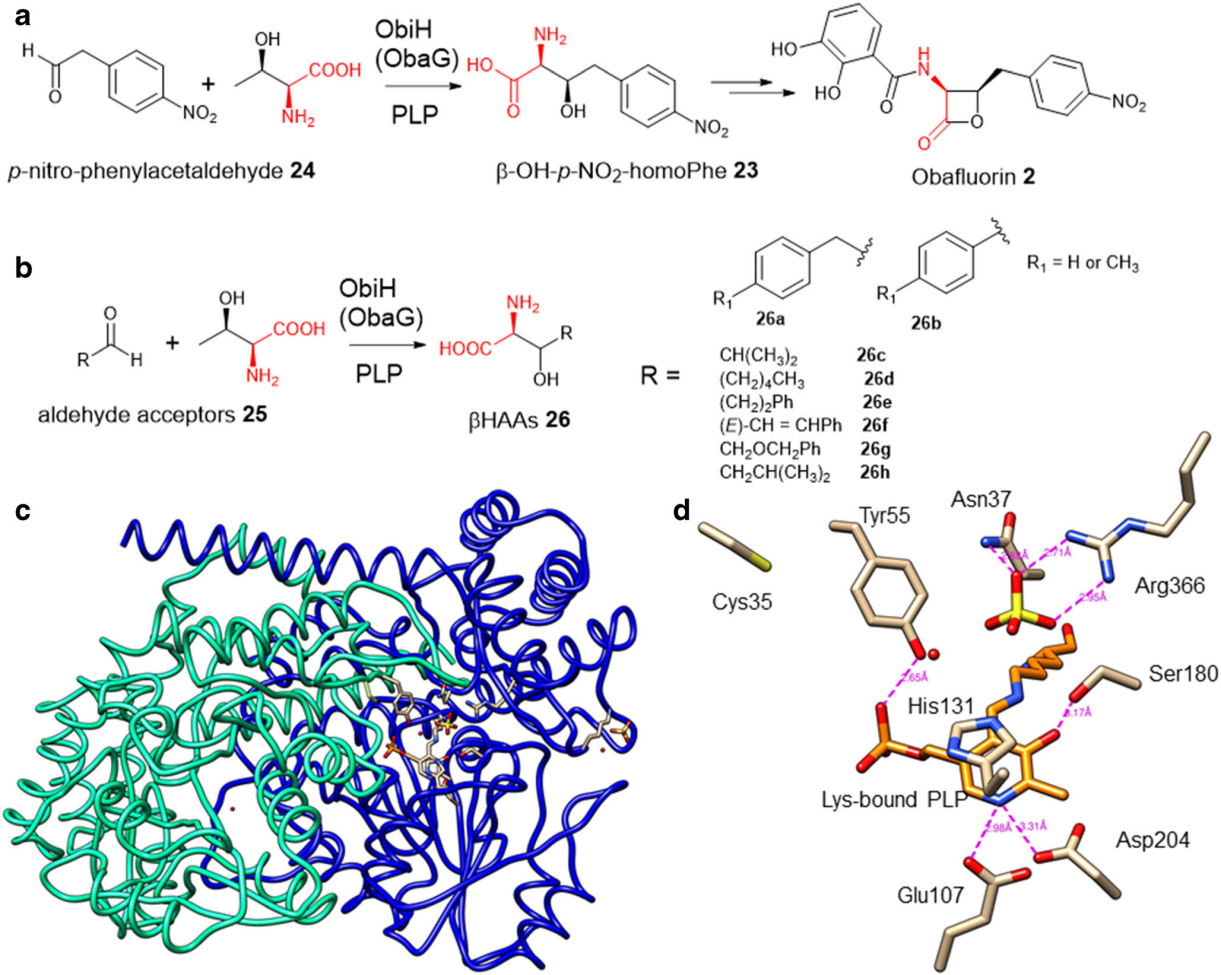
**a** ObiH (ObaG) catalyses a transaldolation between l-Thr and *p*-nitro-phenylacetaldehyde **24** in the biosynthetic pathway of the β-lactone antibiotic natural product, obafluorine **2**. **b** ObiH displays considerable substrate flexibility towards various aryl and aliphatic aldehydes. **c** The structure of ObiH, the first structure of LTTA, indicating that ObiH forms a dimer (PDB access number: 7K34). Individual monomers are coloured in blue (chain A) and cyan (chain B). **d** The active site residues of ObiH are shown in sticks while the Lyn-bound PLP is shown in orange. Hydrogen bonds are shown as pink dashes. The active site of ObiH would allow targeted engineering approaches to further improve the efficiency of the transaldolation reactions catalysed by LTTAs in a broad context.

ObiH displays considerably good substrate promiscuity towards aldehyde acceptors, phenylacetaldehydes, various aliphatic and extended aryl aldehydes as well as a range of aromatic aldehydes (Kreitler et al. 2019). For example, incubation of phenylacetaldehyde with ObiH led to a single stereoisomer, (2*S*)-amino-(3*R*)-hydroxy-4-phenylbutanoate **26a**, while the reaction with benzaldehyde was less efficient leading to two products of l-*threo* and l-*erythreo*-phenylserine **26b** in a ratio of 1:2 and poor overall yield (>20%) (Fig. 5b) (Kreitler et al. 2019). Assay of an α-branched isobutyraldehyde with ObiH resulted in the production of a single stereoisomer, (2*S*,3*R*)-β-hydroxy-Leu **26c**, a βHAA that is difficult to be chemically constructed involving multiple methods (Fig. 5b) (Kreitler et al. 2019). These results suggested that ObiH and its homologues hold a potential for high diastereoselectivity depending on the substrate aldehydes for the synthesis of enantiomerically pure l-*threo*-β-hydroxy-α-amino acids.

The first crystal structure of LTTA was reported in 2021 (Kumar et al. 2021). ObiH (PDB number: 7K34) crystallized as a homodimer (Fig. 5c), with an extension of the C-terminus forming the dimer interface, consistent with other members of the fold-type I superfamily of PLP-dependent enzymes (Eliot and Kirsch 2004). The ObiH active site lies at the dimer interface, with most of the key residues contributed from a single unit (Kumar et al. 2021). Sequence alignment with other LTTAs and SHMTs and structural information revealed that Lys234, Arg366, His131, Tyr55 and Asp204 in the case of ObiH (Fig. 5d) are highly conserved and are in the close proximity of the PLP cofactor in the active site (Kumar et al. 2021). The pyridine ring of PLP is stacked with His 131, and the PLP phosphate is buried with extended H-bond networks, including two to Tyr55 and Asn268 from the partner subunit. Asp204 forms hydrogen bonds to the pyridine nitrogen of PLP, thereby increasing the electrophilicity of pyridinium moiety. Mutational analysis indicated that Asp204 is essential for the catalysis (Kumar et al. 2021).

### LTTA from the biosynthetic pathway of thioheptose nucleoside antibiotic natural products

**Fig. 6:**
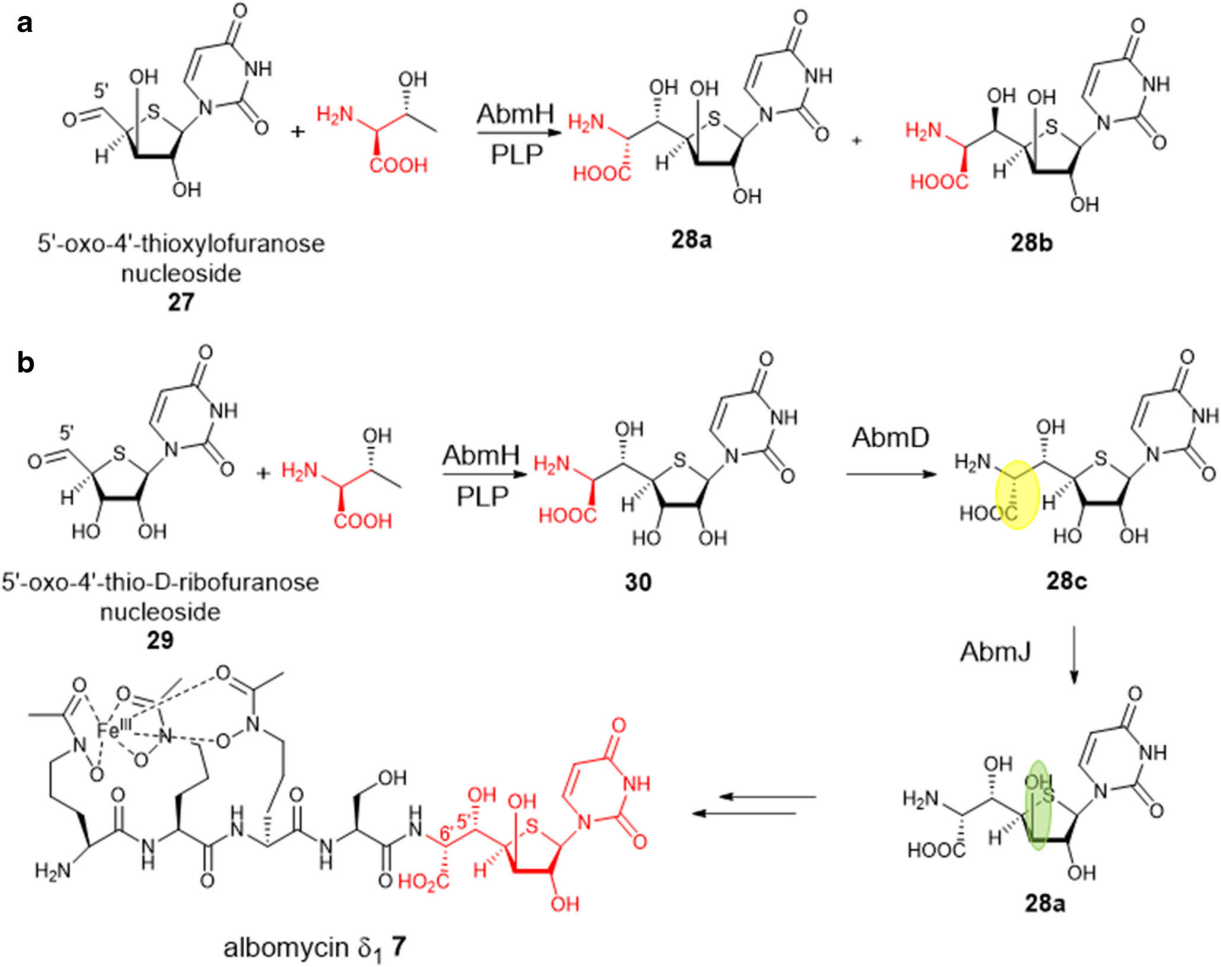
AbmH catalyses the transaldolation reaction between l-Thr and a very unusual thioribofuranose aldehyde substrate, followed by two epimerization events catalysed by a radical SAM AmbJ and PLP-dependent epimerase AmbD to finally install the thiopentose moiety in albomycins

Albomycins **7** are sulphur-containing sideromycin antibiotics, which have intriguing structures consisting of a 6′-amino-4′-thioheptose nucleoside **27** and an iron-chelating ferrichrome siderophore that are linked via amide linkage to an l-Ser residue (Fig. 6). Unlike lipopeptidyl nucleoside antibiotics such as A90289s that contain d-ribose and l-amino acid residues in the high-carbon nucleoside scaffold, albomycins has the d-configuration (6′*R*) of the α-amino acid and the d-*xylo*-configuration (3′*R*) of the furanose ring in the thioheptose core which is rare occurrence found in nature. Bioinformatics analysis suggested that the corresponding BGC of albomycins contain an *orf* (*amb*H), the gene product of which encodes an LTTA-like enzyme that was proposed to catalyse the transaldol reaction of l-Thr and 5′-oxo-4′-thioxylofuranose nucleoside **27** to form the C′5-C′6 bond in **28** (Fig. 6a).

However, biochemical analysis demonstrated that 5′-oxo-d-thioribose nucleoside **29** is the *bona fide* substrate of recombinant AmbH (Ushimaru and Liu 2019). AmbH catalyses a regio- and stereo-selective transaldol reaction between l-Thr and **29** to yield the thioheptose core with a d-ribofuranose ring and an l-amino acid moiety **30**, followed by the epimerization of the l-amino acid to generate d-amino acid catalysed by another PLP-dependent racemerase/epimerase AmbD to provide **28c**. The d-thioribose ring to d-xylofuranose counterpart **28a** is likely to be mediated by the radical SAM epimerase AmbJ based on the gene deletion and complementation experiments (Fig. 6b). The latter two epimerization events catalysed by AmbD and AmbJ appear to be essential for conferring the antimicrobial activity of albomycins despite no major structural alterations (Ushimaru and Liu 2019). AmbH displays considerable substrate plasticity toward 3′*R*-epimer of **29**, d-xylose-based nucleoside aldehyde **27** (Fig. 6a), but the enzymatic products lost stereoselectivity, providing a mixture of stereoisomers (Ushimaru and Liu 2019). The insertion of a sulphur atom into the d-ribofuranose ring during the biosynthesis of albomycins has remained to be determined.

## Discovery of LTTA homologue as biocatalysts

The discovery of the single-domain LTTAs in the biosynthesis of natural products facilitates a conserved genomic approach with the aim of identifying new LTTA homologues. Given the importance of βHAAs and difficulty of constructing enantiomer of βHAAs, there has been a considerable attraction to utilising LTTA homologues as biocatalysts to provide a single-step synthesis of βHAAs under the mild conditions with high stereoselectivity.

**Fig. 7:**
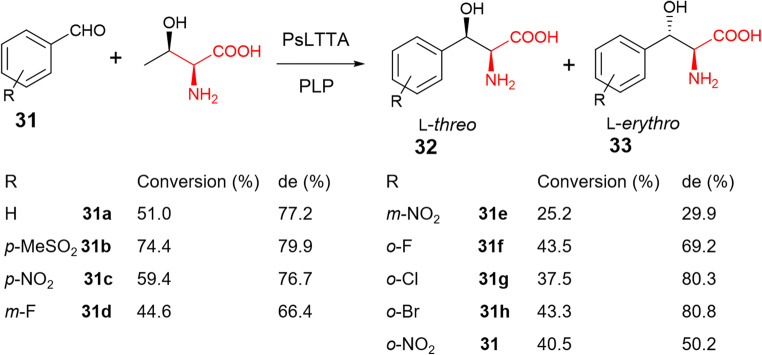
A ObiH homologue, PsLTTA, catalyses a promiscuous transaldolation to generate various phenylserine derivatives

This is the case of PsLTTA from a *Pseudomonas* strain, which has 99% sequence identity to ObiH (Xu et al. 2019). Biochemical analysis demonstrated that PsLTTA accepts l-Thr as the donor substrate among Thr isomers but consumes a wide range of aromatic aldehydes with various substituents in *o*, *p*, *m* position in the aromatic ring as acceptors (Fig. 7). Among these aldehyde acceptors, PsLTTA is able to mediate *p*-methylsulfonyl-benzaldehyde **31b** and l-Thr to produce l-*threo*-*p*-methylsulfonylphenylserine **32** with a high conversion rate and a high de value. It is worth noting that **32** is the key intermediate for synthesis of thiamphenicol and florfenicol. In an optimised condition, the whole cell containing overexpressed recombinant PsLTTA provided an excellent conversion of 67.1% with almost perfect stereoselective ratio of 94.5%. Synthesis of **32** was also conducted in a 100-mL scale of whole-cell biotransformation, first demonstrating that PsLTTA could be utilised as a robust biocatalyst for one-step preparation of βHAAs.

## Directed evolution of mutated LTTA homologue with enhanced capacity

One-step stereoselective enzymatic synthesis of βHAAs has already attracted a considerable attention as this method offers better atomic economy under mild conditions with few by-products generated compared to chemical synthesis. Although PsLTTA showed the potential to catalyse the asymmetric synthesis of **32**, the overall productivity was moderate, preventing the enzyme to be used as viable biocatalysts.

There are two factors that influence the catalytic efficiency. Firstly, PsLTTA catalyses a reversible reaction depending on the concentrations of substrates and products. The accumulation of acetaldehyde may inhibit the forward transaldolation reaction. Secondly, the aldehyde donors are not the natural substrate of PsLTTA, resulting in low conversion and possibly less stereoselectivity. Therefore, it would be desirable to create an enzymatic system that consists of an engineered PsLTTA with an improved activity towards aldehyde acceptors and other enzymes to eliminate the accumulation of acetaldehyde.

**Fig. 8:**
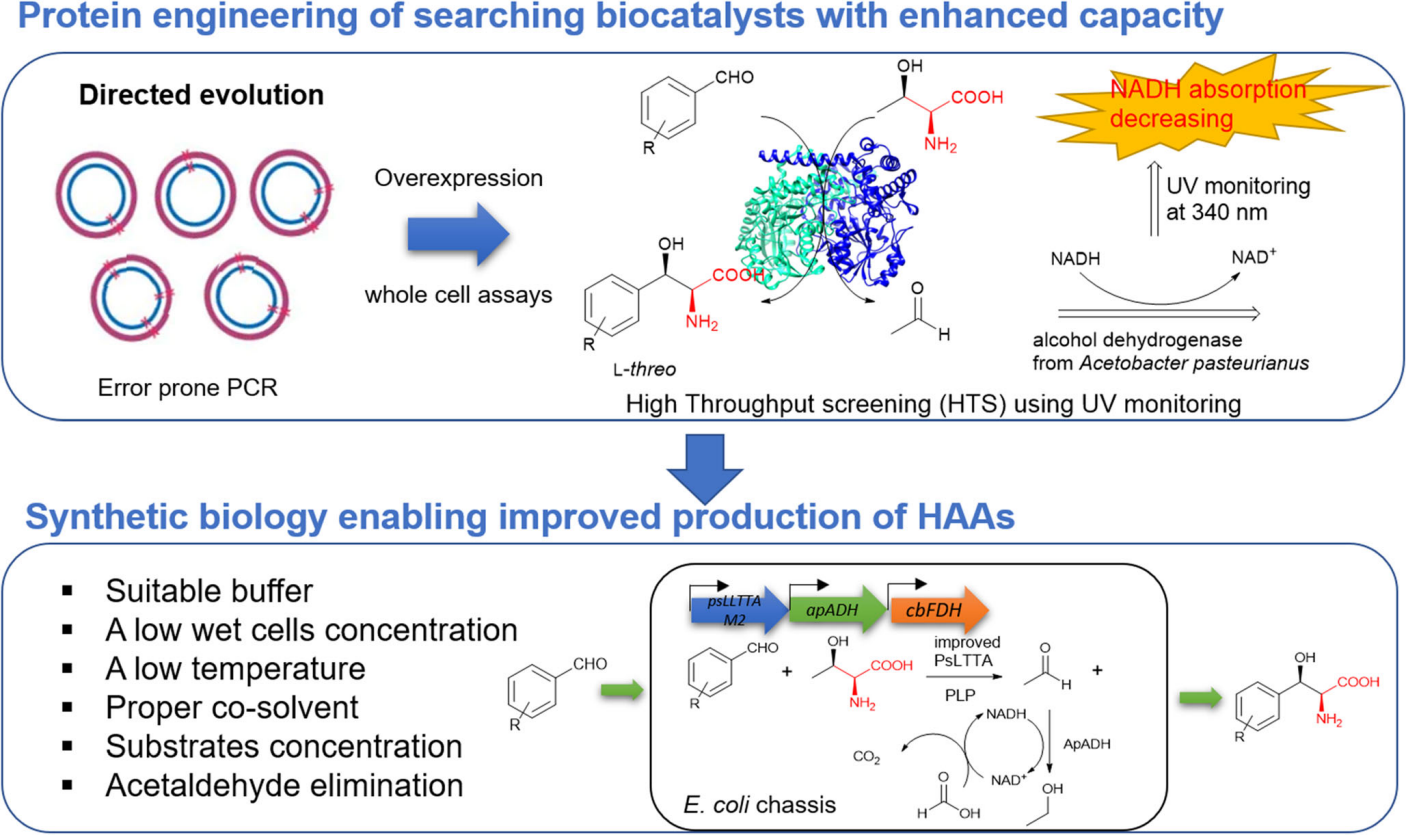
Directed evolution and high throughput screening development enable the discovery of a PsLTTA variant with higher enantioselectivity and productivity, followed by synthetic biology approach to create an engineered *E. coli* chassis for βHAA production.

Directed evolution has been proven to be a powerful method for improving biocatalyst’s activities (Packer and Liu 2015). The key to apply directed evolution is to develop a high throughput screening (HTS) method (Ye et al. 2018). To this end, Xu et al. developed a coupled enzymatic reaction that can be used as the HTS (Xu et al. 2020). Firstly, they applied error-prone PCR method. Overexpression of PCR-mutated genes in *E. coli* and the whole-cell assays in 96-well plate with the reaction cocktail including PLP, aldehyde acceptor and l-Thr were carried out. In the reaction supernatants, alcohol dehydrogenase from *Acetobacter pasteurianus* (ApADH) was added to transform acetaldehyde to ethanol and convert NADH to NAD^+^, the latter activity of which can be assessed by monitoring the reduction of NADH at 340 nm (Fig. 8). More than 4000 colonies were screened. As a result, the double-point PsLTTA variant (PsLLTTAM2) where Asn35 and Cys57 were changed to Ser and Asn, respectively, was found to have the maximal enhanced production of the target βHAA with about 2.1-fold increase compared to wild-type PsLTTA in the whole-cell assays. Subsequently, PsLLTTAM2 was purified for biochemical characterization. The apparent catalytic efficiency (*k*_cat_/*K*_m_) of PsLLTTAM2 was 7.2-fold higher than the one for wild-type PsLLTTA. It is worth noting that such two mutations in PsLLTTA are consistent with the assessment of the structure of ObiH (Kumar et al. 2021) that Asn35 is in the active site of the enzyme and that Cys57 is within a highly mobile loop.

To further improve the yield of βHAA produced by PsLLTTAM2, Xu et al (Xu et al. 2020) developed a regeneration cycle of NADH coupling with the third enzyme, formate dehydrogenase from *Candida boidinii* (CbFDH), to convert NAD^+^ back to NADH using formic acid as substrate (Fig. 8). Such an acetaldehyde elimination system significantly improved the titre of **32** by 4-fold (61.3 mM) compared to the one without the acetaldehyde elimination system in the whole-cell studies (Xu et al. 2020). Finally, an engineered *E. coli* chassis was generated, which contains three genes, *psLLTTAM2*, *cbFDH* and *apADH*. The titre of **32** from this engineered strain was further up to 101.7 mM, 8.8-fold increase compared to the single system of PsLLTTA. The benefit for this synthetic biology approach is that the biotransformation generates ethanol as the by-product. However, the high concentration of accumulated ethanol may inhibit the growth of this engineered *E. coli*, resulting in low productivity of βHAAs in turn in the industrial setting. Therefore, it may be worthy considering using other microbial chassis with high tolerance of ethanol production in the future development.

## Conclusion and future perspectives

Although the first LTTA was discovered almost two decades ago, the catalytic potential of this group of enzymes was only assessed during the last decade, coincident with the advanced genome sequencing technology. Much is still needed to expand the scope of LTTA biocatalysts for asymmetric synthesis of industrially important βHAAs. It is exciting to fully use the existing enzyme inventory to accommodate new chemistries for biotechnologically viable βHAA synthesis. Within this limited pool of identified enzymes, some LTTAs display great substrate flexibility toward aldehyde acceptors with considerably good stereoselectivity. However, the overall productivity of the βHAAs is not yet desirable as a viable biocatalyst. As a complement to this, we should also turn our attention to protein engineering to enhance the activities of the promising enzyme candidates. This was exemplified by the recent HTS method which has set a framework for directed evolution of LTTAs (Fig. 8).

**Fig. 9:**
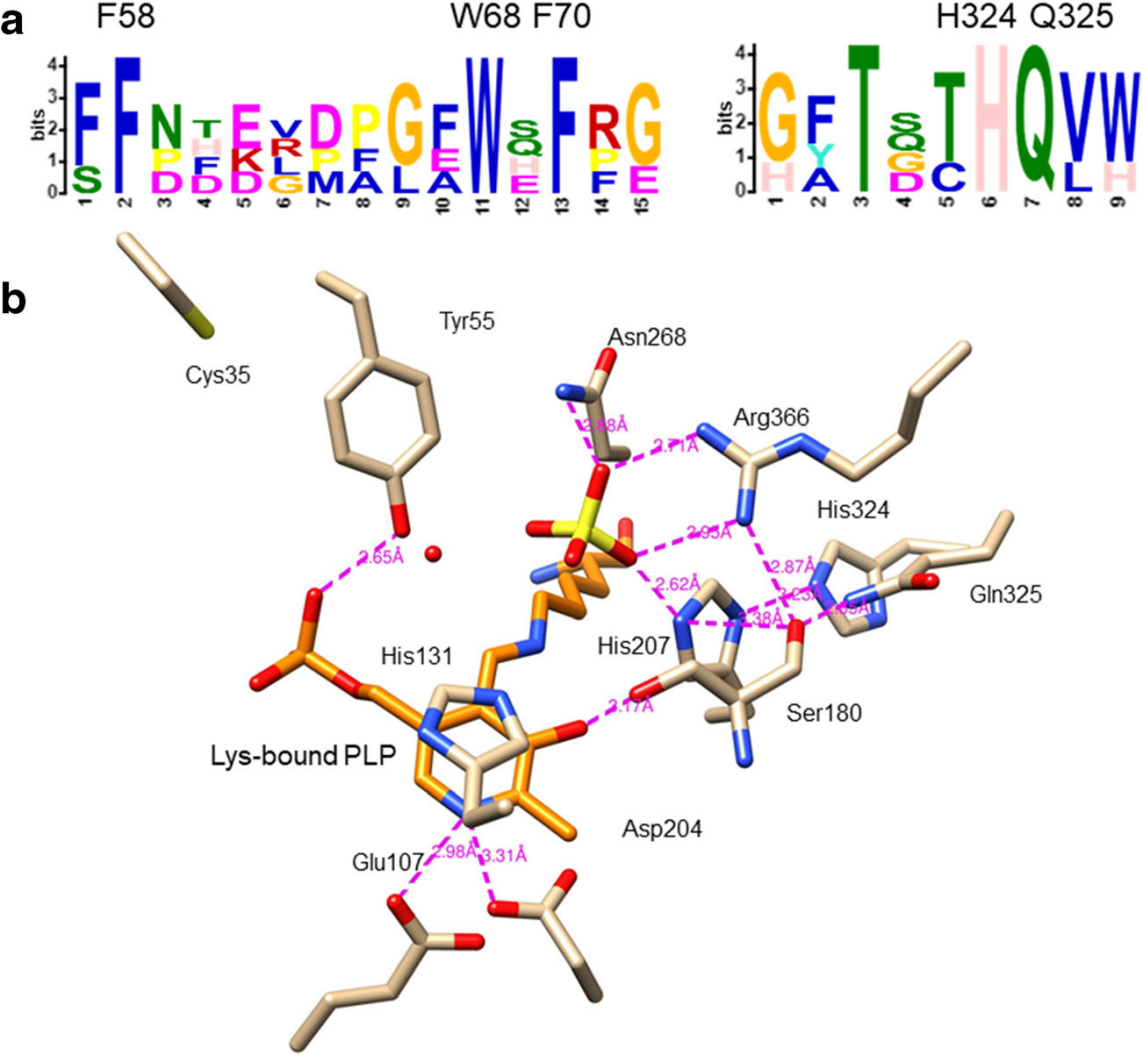
**a** Protein motif analysis suggested that LTTAs contain two unique aa motifs compared to *E. coli* SHMT enzyme. Some of aa residues are highly conserved among LTTAs. **b** The hydrogen bond network formed among H324 and Q325 with the residues, H207 and R366, in the active site of ObiH is shown as pink dashes. Lys-bound PLP is shown in orange

Another potential exciting development is to turn our attention to nature for inspirations by discovering new βHAA-containing entities through natural product programmes and new LTTAs with broad substrate specificity, better kinetics, high regio- and stereo-selectivity and good tolerance towards reaction conditions (i.e. organic solvents, temperatures) by genomic mining strategy (Fig. 8). It should be noted that the majority of LTTAs was identified from two bacterial species, *actinomycetes* and *pseudomonas*, in the current enzyme inventory. Protein motif analysis (Bailey et al. 2009) combined with the available structure data of ObiH (Kumar et al. 2021) suggested that LTTAs contain unique conserved motifs, including F58, W68 and F70 in the mobile loop of ObiH, and H324 and Q325 which form extensive hydrogen bond network with the residues, H207 and R366, in the active site of ObiH (Fig. 9). This information could serve as guidance for future LTTA mining. Therefore, efforts directed towards LTTA candidates from other microbial genus will certainly offer a promising opportunity of discovering a biotechnologically viable biocatalyst for βHAA synthesis.
